# The influence of dietary energy and zinc source and concentration on performance, trace mineral status, and gene expression of beef steers

**DOI:** 10.1093/tas/txaa207

**Published:** 2020-11-14

**Authors:** Remy N Carmichael-Wyatt, Olivia N Genther-Schroeder, Stephanie L Hansen

**Affiliations:** Department of Animal Science, Iowa State University, Ames, IA

**Keywords:** beef cattle, dietary energy, growth, metallothionein, zinc

## Abstract

The objective of this study was to determine the effects of increased supplemental Zn from differing sources on growth performance of steers fed diets differing in net energy. Angus steers (*n* = 72, 324 ± 2.1 kg) with Genemax gain scores of 3, 4, or 5 were blocked by BW and stratified by Genemax gain score into 12 pens of 6 steers each for 158 d. Pens were randomly assigned to 1 of 3 Zn treatments (ZNTRT): 1) control (no supplemental Zn, analyzed 33 mg Zn/kg DM; CON); 2) inorganic Zn (CON + 120 mg supplemental Zn/kg DM as ZnSO_4_ for entire trial; INZN); or 3) 120 mg supplemental Zn/kg DM as Zn-amino acid complex (Availa-Zn; Zinpro, Eden Prairie, MN) for first 60 d, then a blend of ZnSO_4_ and Zn-AA complex (CON + 60 mg supplemental Zn/kg DM as ZnSO_4_ + 60 mg supplemental Zn/kg DM as Zn-amino acid complex) for the remainder of the trial (ZNBLD). Two dietary energy strategies (ENERGY) were formulated to reach ADG rates of 1) 1.6 kg/d (LE) or 2) 2.0 kg/d (HE) utilizing a 3 × 2 factorial arrangement (12 steers/treatment). All steers were fed LE for a 60 d growing period, then pens were randomly assigned to ENERGY treatments fed the remaining 91 d. Day 60 BW tended to be greater (*P* = 0.07) in steers receiving supplemental Zn vs. CON. Liver Cu was decreased in Zn supplemented steers vs. CON (*P* = 0.02). Liver Zn concentrations on d 56 did not differ for Zn vs. CON (*P* = 0.22) nor were there differences due to Zn source (*P* = 0.98). There were or tended to be ZNTRT × ENERGY effects for d 67–90 ADG and G:F (*P* ≤ 0.01), and d 122 BW and d 90–122 G:F (*P* ≤ 0.10) driven by improved performance for ZNBLD-HE over ZNBLD-LE, while ENERGY within CON and INZN did not differ. Day 90–122 ADG, overall ADG and overall G:F was greater (*P* ≤ 0.02) and d 67–90 G:F tended to be greater (*P* = 0.10) for HE vs. LE. No ZNTRT × ENERGY or ZNTRT effects were detected for HCW, REA, BF, KPH, MS, or YG (*P* ≥ 0.37) while HE increased HCW, BF, MS, and YG compared with LE (*P* ≤ 0.05). In the liver, ZNTRT affected d 97 MT1A expression (*P* = 0.03) where INZN was greater than ZNBLD or CON (*P* ≤ 0.02), while ZIP14 was unaffected due to ZNTRT, ENERGY, or the interaction (*P* ≥ 0.39). Supplying supplemental Zn as ZNBLD during the transition period appeared to improve performance measures, but no final performance advantages were noted due to increased supplemental Zn, regardless of source. Additionally, differences in liver MT1A expression may indicate differing post-absorptive metabolism between Zn sources.

## INTRODUCTION

The trace mineral Zn supports a variety of metabolic processes in mammals as an important constituent in pathways such as DNA and protein synthesis (Cousins et al., 2006). Current NASEM (2016) recommendations for Zn (30 mg Zn/kg DM) were established more than 40 yr ago. However, since 1977 cattle ADG has increased approximately 44% (Capper, 2011), to some degree due to growth-promoting technologies. When cattle are supplemented with the β-adrenergic agonist (β-AA) ractopamine hydrochloride for the last 33 d on feed a 24% increase in ADG can be achieved (Avendaño-Reyes et al., 2006). Previous work has shown that increasing supplemental Zn (as a blend of organic and inorganic sources) to well over NASEM (2016) recommendations has increased the growth of ractopamine hydrochloride-fed steers, suggesting a greater need for dietary Zn when cattle experience rapid growth (Genther-Schroeder et al., 2016a, 2016b). Follow-up work by Carmichael et al. (2018) using the same dietary Zn and β-adrenergic agonist supplementation strategies noted N retention was increased 5% by ractopamine hydrochloride, and was also improved 4% due to supplemental Zn. Indeed, Zn has been shown to be integral to N retention in other species, possibly due to its role in protein synthesis (Oberleas and Prasad, 1969; Somers and Underwood, 1969; Greeley et al., 1980). It is well known that increasing dietary net energy increases cattle growth rate (Kleiber, 1961; Lofgreen and Garrett, 1968) and while ractopamine hydrochloride causes a rapid increase in growth, Zn may be important to support rapid growth in cattle not receiving a β-AA. Therefore, the objective of this study was to determine the effects of increased supplemental Zn on growth performance of steers fed diets differing in net energy not receiving a β-AA. The hypothesis was that steers experiencing greater growth rates induced by the high energy diet would further benefit from increased supplemental Zn compared with those receiving the low energy diet.

## MATERIALS AND METHODS

All procedures and protocols were approved by the Iowa State University Institutional Animal Care and Use Committee (8-15-8073-B).

### Experimental Design

A total of 72 Angus steers (324 ± 2.1 kg) from a single source with Genemax (Zoetis, Parsippany, NJ) gain scores of 3, 4, or 5, indicating a predicted genetic value belonging in the top 60% for growth potential of tested Angus cattle (Zoetis, 2012), were utilized in this study. Approximately 30 d before the beginning of the study, steers received a booster vaccine for Bovine Viral Diarrhea Virus type 1 and II (Bovi-Shield Gold, One Shot, Zoetis) and received a broad coverage dewormer pour-on solution dose (Dectomax, Zoetis). Steers were blocked by BW and stratified by Genemax gain score into 12 pens of 6 steers each. Pens were randomly assigned to 1 of 3 Zn treatments (ZNTRT) for the entirety of the trial: 1) control (no supplemental Zn, analyzed 33 mg Zn/kg DM; CON); 2) inorganic Zn supplementation (CON + 120 mg supplemental Zn/kg DM as ZnSO_4_; INZN); or 3) blend of supplemental Zn sources (ZNBLD) where steers received 120 mg supplemental Zn/kg DM as Zn-amino acid complex (Availa-Zn; Zinpro, Eden Prairie, MN) for the initial growing period of 60 d, then switched to a blend of ZnSO_4_ and Zn-AA complex [CON + 60 mg supplemental Zn/kg DM ZnSO_4_ + 60 mg supplemental Zn/kg DM as Zn-amino acid complex] for the remainder of the trial. These supplemental Zn concentrations were chosen for the current study to coincide with previous research completed by Genther-Schroeder et al. (2016a, 2016b) and Carmichael et al. (2018).

The trial consisted of two phases: an initial growing period where all steers received a low-energy (LE) diet for 60 d, followed by a 91 d finishing period where half the steers transitioned to a high-energy (HE) diet while half remained on LE. The two dietary energy (ENERGY) strategies were formulated to reach targeted growth rates of 1) 1.6 kg ADG (LE) or 2) 2.0 kg ADG (HE). Diet composition and analysis are shown in Table 1. For the initial 60 d (growing period) of the study, all steers (*n* = 24 steers/ZNTRT) were fed the LE diet. Beginning on d 60, 2 pens per ZNTRT (one pen from each weight block) was assigned to HE and transitioned for 7 d. Feed delivery on the first day of transition was such that steers received a similar amount of dry matter (DM) as the day before when all were receiving the LE diet. Adjustments in feed delivery over the rest of the transition period were made according to the previous day's bunk score. Following the transition, all steers were fed their respective treatment combination for a 91 d finishing period (*n* = 12 steers/treatment combination).

**Table 1.** Diet ingredient composition and nutrient content (% DM basis)

	LE^*a*^			HE^*a*^		
Ingredient	CON^*b*^	INZN^*b*^	ZNBLD^*b*^	CON^*b*^	INZN^*b*^	ZNBLD^*b*^
Cracked corn	22.3	22.3	22.3	41.8	41.8	41.8
Corn silage	40	40	40	26.2	26.2	26.2
Modified distillers grains	22	22	22	22	22	22
Dried distillers grains^*c*^	5	5	5	5	5	5
Hay	5.7	5.7	5.7	0	0	0
Micronutrients and carrier^*d*^	5	5	5	5	5	5
Calculated components^*e*^						
NEm, Mcal/kg	1.89	1.89	1.89	2.05	2.05	2.05
NEg, Mcal/kg	1.25	1.25	1.25	1.38	1.38	1.38
Analyzed components						
Crude protein, %	15.3	15.3	15.3	16.3	16.3	16.3
NDF, %	31.1	31.1	31.1	21.8	21.8	21.8
Cu	15	15	15	15	15	15
Fe	95	90	91	94	78	91
Mn	33	33	33	33	33	32
Zn	33	133	143	33	146	143

^*a*^LE diet (calculated to target ~1.6 kg ADG); HE diet (calculated to target ~2.0 kg ADG).

^*b*^Control (CON) received no supplemental Zn (diets analyzed 33 mg Zn/kg DM); Inorganic Zn (INZN) diet received 120 mg supplemental Zn/kg DM (CON + 120 mg Zn/kg DM as ZnSO_4_); Zinc blend (ZNBLD) diet received 120 mg supplemental Zn/kg DM (CON + 60 mg Zn/kg DM as ZnSO_4_ and 60 mg Zn/kg DM as Availa-Zn [Zinpro Corporation, Eden Prairie, MN]).

^*c*^Dried distillers grains alone or as carrier for INZN and ZNBLD premix.

^*d*^Basal includes dried distillers grains with solubles as carrier and micronutrients to provide to total diet (DM basis); limestone (1.4%), Rumensin (0.0135%), urea (0.3%), and salt (0.31%). Trace minerals and vitamins provided per kilogram of total diet DM: 0.15 mg Co (cobalt carbonate), 10 mg Cu (copper sulfate), 20 mg Mn (manganese sulfate). 0.1 mg Se (sodium selenite), 0.5 mg I (calcium iodate), Vitamin A 2,200 IU (Rovimix A 1000 [1,000 kIU/g], DSM, Parsippany, NJ), and Vitamin E 25 IU (Rovimix E50 [50 kIU/g], DSM, Parsippany, NJ).

^*e*^Net energy values calculated from NASEM (2016).

During the entire trial period (158 d) all steers were fitted with unique electronic identification tags and were housed in pens equipped with GrowSafe bunks (GrowSafe Systems Ltd., Airdrie, Alberta, Canada) to collect daily individual as-fed intake. Steers were fed once daily at 8:00 a.m. and provided ad libitum access to feed and water. On d 0 all steers were implanted with Component TE-IS with Tylan (80 mg trenbolone acetate, 16 mg estradiol USP, and 29 mg tylosin tartrate; Elanco Animal Health, Greenfield, IN) and on d 90 all steers were implanted with Component TE-S with Tylan (140 mg trenbolone acetate, 24 mg estradiol USP, and 29 mg tylosin tartrate; Elanco Animal Health). Pre-feeding BW were recorded prior to feeding on d −1, 0, 59, 60, 66, 67, 90, 122, 157, and 158, and a 4% pencil shrink was applied to all BW measurements before calculation of ADG and gain-to-feed (G:F).

Liver and muscle biopsies were collected on d 56, 97, and 153. Liver biopsies were collected using methods established by Engle and Spears (2000). For muscle biopsies, the area was clipped, scrubbed with betadine and 70% ethanol, and injected with 10 mL of 2% lidocaine between the 11th and 12th ribs into the *Longissimus dorsi* muscle. A modified Jamshidi bone marrow biopsy/aspiration needle (8 g × 10 cm needle) was inserted into the injected area to collect muscle tissue (~0.5 g wet basis). The collected tissue was rinsed with 0.1 M phosphate-buffered saline (pH 7.0) to remove any vestigial blood from the sample, placed into an acid-washed container and flash frozen in liquid nitrogen. Muscle tissue was stored at −80 °C until further analysis.

Blood was collected by jugular venipuncture on d 59, 90, 122, and 157 into serum, sodium heparin, K_2_ EDTA Plus, and trace element K_2_ EDTA blood collection tubes (Becton, Dickinson and Company, Franklin Lakes, NJ) for analysis of plasma trace mineral (TM) and various blood metabolites.

At the end of the experiment (158 d), steers were shipped to a commercial abattoir (Iowa Premium, Tama, IA) for slaughter followed by a 48 h chilling period. Personnel from the Hansen laboratory, blinded to treatments, were dispatched to collect carcass data, including hot carcass weight (HCW), *Longissimus dorsi* muscle area (REA) 12th rib fat thickness (BF), kidney, pelvic, and heart fat (KPH), and marbling score (MS), all of which were utilized to calculate yield grade (YG) and quality grade (QG).

### Feed, Tissue, and Blood Analysis

Total mixed ration (TMR) samples of each diet were collected weekly and subsamples were taken to determine DMI. Dry matter of TMR was determined according to AOAC (1990) procedures. Dried TMR were ground through a 2 mm screen (Retsch Zm100 grinder; Glen Mills Inc., Clifton, NJ) and stored until nutrient analysis. Feed efficiency was calculated from the total gain and total DMI determined at each weighing interval.

Inductively coupled plasma optical emission spectrometry (ICP-OES; Optima 7000 DV, Perkin Elmer, Waltham, MA) was used for trace mineral analysis of TMR, plasma, and liver tissue. Dried, ground, and composited TMR were acid digested prior to mineral analysis as described by Pogge et al. (2014). Liver and plasma samples were digested and analyzed for Zn and Cu according to Pogge and Hansen (2013). To verify instrument accuracy a bovine liver standard from the National Institute of Standards and Technology (Gaithersburg, MA) or serum standard (UTAK Laboratories, Inc., Valencia, CA) was utilized and yttrium (PerkinElmer, Waltham, MA) served as an internal standard to account for sample introduction variation. Serum non-esterified fatty acid (NEFA) concentrations were measured by colorimetric assay (HR series NEFA-HR(2) assay kit; Wako Pure Chemical Industries, Ltd., Osaka, Japan; inter-assay CV of 9.2%). Serum urea nitrogen (SUN) concentrations were determined by colorimetric assay (B551; TECO Diagnostics, Anaheim, CA; inter-assay CV of 9.8%). Heparinized plasma was analyzed to determine plasma insulin-like growth factor-1 (IGF-1) concentrations in a commercial ELISA kit (SG100; R&D Systems, Minneapolis, MN; inter-assay CV of 1.9%) shown to have 100% cross-reactivity with bovine IGF-1 (Moriel and Arthington, 2013).

Liver and muscle samples for mRNA extraction were kept frozen with liquid nitrogen while ground with a mortar and pestle. Extraction of mRNA was conducted using the Trizol (Life Technologies, Carlsbad, CA) method and the RNeasy Mini Kit (Qiagen GmbH, Qiagen Strasse 1, Hilden, Germany) per manufacturer’s instructions. A Qubit 4 Fluorometer (Invitrogen, ThermoFisher Scientific, Waltham, MA) was used to quantify and measure the integrity and quality of RNA. For each sample, 0.5 μg was reverse transcribed using the Superscript III kit (ThermoFisher Scientific) per the manufacturer’s instructions. Complementary DNA samples were prediluted (1:10) and 2μL were combined with 20μL of a master mix containing SYBR Green fluorescent dye (Life Technologies LTD, Warrington, UK), forward and reverse primers (Integrated DNA Technologies, Coralville, IA) and RNAse free water for gene expression analysis. Utilizing quantitative real-time PCR, samples were analyzed for RNA abundance of 40s Ribosomal Protein 9 (Rps9; 5′–3′ CCTCGACCAAGAGCTGAAG; 3′–5′ CCTCCAGACCTCACGTTTGTTC; annealing temperature 52 °C; Janovick-Guretzky et al., 2007), Zrt- and Irt-like protein 14 (ZIP14; 5′–3′ AGGCTCCTGCTCTACTTC; 3′–5′ AGCGTCTCAGAGGTATAATG; annealing temperature 56 °C; Hansen et al., 2010), and Metallothionein 1A (MT1A; 5′–3′ ATGGACCCGAACTGCTCCTGC; 3′–5′ GCGCAGCAGCTGCACTTGTCCG; annealing temperature 59 °C; Fry et al., 2013) within liver and muscle tissue. Melting temperature during the 40 amplification cycles was 94 °C and resulting cycle threshold (Ct) values were normalized to the abundance of RPS9. The resulting values for gene expression are ΔCt, calculated by subtracting Ct values of RPS9 from the Ct value of the gene of interest. Relative fold change expression of the Metallothionein 1A and Zrt- and Irt-like protein 14 genes were calculated by subtracting the average of CON-LE (ΔΔCT) from each ΔCt value and raising to the power of 2 (2^−ΔΔCT^).

### Statistical Analysis

Data were analyzed as a randomized complete block design. Performance, DMI, and liver TM data for the initial 60 d growing period were analyzed using the Mixed procedures of SAS (SAS Institute Inc., Cary, NC), with steer as the experimental unit (*n* = 24 per ZNTRT). The model included the fixed effects of ZNTRT and block. Two single degrees of freedom contrasts were constructed for the growing period: 1) no supplemental Zn vs. supplemental Zn as both INZN and ZNBLD and 2) INZN vs. ZNBLD. Data collected following d 67 (finishing period) were analyzed as a 3 × 2 factorial arrangement using the Mixed procedure of SAS. The model included the fixed effects of ZNTRT, ENERGY, block, and the interaction of ZNTRT and ENERGY; steer was the experimental unit (*n* = 12/treatment combination) for all analyses. Finishing period plasma and IGF-1, SUN, and serum NEFA data were analyzed as repeated measures with date as the repeated effect. The compound symmetry variance structure for repeated measures was selected to achieve the lowest Akaike information criterion value for all repeated measures analysis and d 60 IGF-1 concentrations were used as a covariate for IGF-1 repeated measures. The Shapiro–Wilk test of normality was utilized and NEFA and finishing period G:F data were normally distributed by log transformation; treatment means and SEM shown are reverse transformed. Outliers were determined using Cook’s D and removed if Cook’s D ≥ 0.5. Due to health reasons unrelated to treatment one steer from ZNBLD-HI was removed from analysis following d 67. Significance was declared at *P* ≤ 0.05 and tendencies identified if *P* ≥ 0.06 and ≤0.10. Tabular values reported reflect the least square means and the PDIFF statement in SAS was utilized to determine pairwise differences.

## RESULTS

### Growing Period Performance

Data for the initial 60 d growing period when all steers received the LE diet are shown in Table 2. No differences were detected for DMI, ADG, or G:F when comparing Zn vs. CON (*P* ≥ 0.12) during this period, nor were there differences due to Zn source (*P* ≥ 0.20). However, d 60 BW tended to be greater in steers receiving supplemental Zn vs. CON (*P* = 0.07). Liver Cu was decreased in Zn supplemented steers vs. CON (*P* = 0.02) while no differences were detected between Zn sources (*P* = 0.48). Liver Mn was not different between supplemental Zn and CON (*P* = 0.16) but was lesser in INZN when compared with ZNBLD (*P* = 0.01). Day 56 liver Zn concentrations did not differ for Zn vs. CON (*P* = 0.22) nor were there differences due to Zn source (*P* = 0.98).

**Table 2.** Dietary Zn influence on initial 60 d growing period performance and liver trace mineral concentrations when all steers received the low energy diet

Item	CON^*a*^	INZN^*a*^	ZNBLD^*a*^	SEM	C vs. Z^*b*^	ZB vs. ZS^*b*^
Steers (*n*)	24	24	24			
Dry matter intake, kg/d	9.11	9.19	9.25	0.237	0.70	0.84
BW^*c*^, kg						
d 0	323	324	326	2.1	0.45	0.72
d 60	429	437	436	3.4	0.07	0.91
ADG^*c*^, kg	1.76	1.87	1.84	0.049	0.12	0.69
Gain to feed^*c*^	0.196	0.201	0.205	0.0062	0.38	0.69
Liver d 56, mg/kg DM						
Cu	417	322	350	27.2	0.02	0.48
Mn	9.3	7.9	9.5	0.330	0.16	0.01
Zn	111	119	118	5.1	0.22	0.98

^*a*^Control (CON) received no supplemental Zn (diet analyzed 33 mg Zn/kg DM); Inorganic Zn (INZN) was CON + 120 mg supplemental Zn/kg DM as ZnSO_4_); Zinc blend (ZNBLD) was (CON + 120 mg supplemental Zn/kg DM as Zn-amino acid complex [Availa-Zn; Zinpro Corporation, Eden Prairie, MN]).

^*b*^Contrasts: C vs. Z = CON vs. ZNBLD and INZN; ZB vs. ZS = ZNBLD vs. INZN.

^*c*^All weight values include 4% pencil shrink in calculations.

### Finishing Period Performance

Effects of ZNTRT and ENERGY strategies on steer performance during the finishing period are shown in Table 3. No ZNTRT × ENERGY effects were noted for DMI, d 67, 90 and final BW, or d 90–122, 122–158, and overall finishing period ADG, or d 122–158 or overall G:F (*P* ≥ 0.21). However, there were or tended to be ZNTRT × ENERGY effects for d 67–90 ADG and G:F (*P* ≤ 0.01), and d 122 BW and d 90–122 G:F (*P* ≤ 0.10). Collectively, these interactions are driven by improved performance during this time by ZNBLD-HE over ZNBLD-LE, while within CON and INZN, HE and LE did not differ for these measures. No ZNTRT effects were noted for interim or overall DMI, BW, ADG, or G:F during the finishing period (*P* ≥ 0.15). An ENERGY effect was detected where HE was greater than LE for d 90–122 ADG, overall ADG and overall G:F (*P* ≤ 0.02), and tended to be greater for d 67–90 G:F (*P* = 0.10). No ENERGY effects were detected for DMI or d 67, 90, or 122 BW, or d 122–158 ADG and d 122–158 G:F (*P* ≥ 0.11).

**Table 3.** Dietary Zn and energy concentration influence on performance measures during the finishing period

	CON^a^		INZN^a^		ZNBLD^a^			*P*-value		
Item	LE^b^	HE^b^	LE^b^	HE^b^	LE^b^	HE^b^	SEM	ZNTRT	ENERGY	ZNTRT × ENERGY
Steers (*n*)	12	12	12	12	12	11				
Dry matter intake, kg/d										
d 67–90	11.0	10.9	11.3	11.2	11.1	11.3	0.32	0.61	0.94	0.91
d 90–122	11.1	11.1	11.4	11.6	11.3	11.2	0.32	0.43	0.85	0.86
d 122–158	11.5	11.1	11.5	12.1	11.7	11.5	0.42	0.50	0.91	0.47
d 67–158	11.2	11.0	11.4	11.6	11.4	11.3	0.33	0.48	0.96	0.82
BW^c^, kg										
d 67	440	440	449	443	442	448	5.3	0.49	0.98	0.61
d 90	492	488	499	497	494	508	6.1	0.18	0.55	0.26
d 122	546	546	557	557	545	570	6.6	0.15	0.15	0.10
Final, d 158	604	609	614	619	605	629	8.2	0.35	0.11	0.44
ADG^c^, kg										
d 67–90^d ^	2.18^ab^	1.98^b^	2.07^b^	2.24^ab^	2.14^b^	2.54^a^	0.093	0.02	0.11	0.01
d 90–122	1.70	1.81	1.84	1.87	1.61	1.92	0.078	0.34	0.02	0.21
d 122–158	1.61	1.76	1.58	1.72	1.67	1.60	0.104	0.88	0.40	0.49
d 67–158	1.81	1.85	1.82	1.93	1.79	2.02	0.059	0.43	0.01	0.30
G:F^c^										
d 67–90^d ^	0.208^ab^	0.191^b^	0.192^b^	0.208^ab^	0.201^b^	0.236^a^	0.0095	0.04	0.10	0.01
d 90–122^d^	0.151^xy^	0.164^xy^	0.163^xy^	0.162^xy^	0.143^y^	0.172^x^	0.0070	0.72	0.02	0.10
d 122–158	0.141	0.158	0.138	0.142	0.144	0.140	0.0081	0.40	0.51	0.31
d 67–158	0.167	0.171	0.165	0.171	0.163	0.181	0.0045	0.62	0.01	0.24

^*a*^Control (CON) received no supplemental Zn (diets analyzed 33 mg Zn/kg DM); Inorganic Zn (INZN) diet was CON + 120 mg supplemental Zn/kg DM as ZnSO_4_; Zinc blend (ZNBLD) diet was CON + 60 mg supplemental Zn/kg DM as ZnSO_4_ and 60 mg supplemental Zn/kg DM as Zn-amino acid complex (Availa-Zn; Zinpro Corporation, Eden Prairie, MN).

^*b*^LE: low energy diet formulated to target ~1.6 kg ADG; HE: high energy diet formulated to target ~2.0 kg ADG.

^*c*^All weight values include 4% pencil shrink in calculations; ADG, average daily gain; G:F, Gain to feed.

^*d*^Results with differing superscripts are different (a,b; *P* ≤ 0.05) or tend to be different (x,y; *P* ≤ 0.10).

Carcass data are shown in Table 4. A ZNTRT × ENERGY effect was noted for DP (*P* = 0.02) where no effect of ENERGY was detected within cattle receiving CON or ZNBLD, but within INZN, HE tended to dress greater than LE. No ZNTRT × ENERGY or ZNTRT effects were detected for HCW, REA, BF, KPH, MS, or YG (*P* ≥ 0.37). The HE diet increased HCW, BF, MS, and YG compared with LE (*P* ≤ 0.05), but ENERGY had no effect on REA or KPH (*P* ≥ 0.63).

**Table 4.** Dietary Zn and energy concentration influence on carcass characteristics for final 91 d finishing period

	CON^*a*^		INZN^*a*^		ZNBLD^*a*^			*P*-value		
Item	LE^*b*^	HE^*b*^	LE^*b*^	HE^*b*^	LE^*b*^	HE^*b*^	SEM	ZNTRT	ENERGY	ZNTRT × ENERGY
Steers (*n*)	12	12	12	12	12	11				
Carcass characteristics										
HCW^*c*^, kg	380	387	384	394	382	394	5.3	0.56	0.03	0.92
DP^*d*^, %	62.9^xy^	63.7^xy^	62.4^y^	63.8^x^	63.2^xy^	62.7^xy^	0.33	0.52	0.06	0.02
REA^*c*^, cm^2^	85.8	84.5	87.1	87.7	85.8	89.0	1.94	0.38	0.63	0.43
Back fat, cm	1.61	1.80	1.57	1.82	1.50	1.83	0.132	0.96	0.02	0.87
KPH^*c*^, %	2.2	2.2	2.0	2.1	2.1	2.1	0.09	0.48	0.67	0.81
Marbling score^*e*^	442	455	445	470	406	466	17.2	0.46	0.02	0.37
Yield grade	3.0	3.3	2.9	3.3	2.9	3.2	0.18	0.81	0.05	0.98

^*a*^Control (CON) received no supplemental Zn (diets analyzed 33 mg Zn/kg DM); Inorganic Zn (INZN) diet was CON + 120 mg supplemental Zn/kg DM as ZnSO_4_; Zinc blend (ZNBLD) diet was CON + 60 mg supplemental Zn/kg DM as ZnSO_4_ and 60 mg supplemental Zn/kg DM as Zn-amino acid complex (Availa-Zn; Zinpro Corporation, Eden Prairie, MN).

^*b*^LE: low energy diet formulated to target ~1.6 kg ADG; HE: high energy diet formulated to target ~2.0 kg ADG.

^*c*^HCW, hot carcass weight; REA, ribeye area; KPH, kidney, pelvic and heart fat.

^*d*^DP, Dressing percentage; Means with differing superscripts tend to be different (x,y; *P* ≤ 0.10).

^*e*^300, slight; 400, small; 500, modest.

### Finishing Period Liver and Plasma Trace Mineral and Blood Metabolites

Plasma Cu and Zn repeated measures data are shown in Table 5 and d 97 and 153 liver Cu, Mn, and Zn data are shown in Table 6. A ZNTRT × ENERGY effect was noted for plasma Zn (*P* = 0.04) where INZN HE was greater than CON or ZNBLD HE and INZN LE (*P* ≤ 0.03) and ZNBLD LE was greater (*P* = 0.003) and INZN tended to be greater (*P* = 0.06) than CON LE. No interactions with time (*P* ≥ 0.24) were noted for plasma Zn concentrations; however, there was an effect of time (*P* = 0.0001) where concentrations were least on d 60 (1.25 mg/L) relative to d 90, 122, and 157 (1.38, 1.33, and 1.38 mg/L, respectively). No ZNTRT × ENERGY × time, ZNTRT × time, or ZNTRT effects were noted for plasma Cu (*P* ≥ 0.19). However, there was an ENERGY × time effect for plasma Cu (*P* = 0.02) where HE tended to be greater than LE on d 90 and 122 (1.02 vs. 0.93 and 1.03 vs. 0.95 mg/L, respectively; *P* ≤ 0.08) while HE and LE did not differ on d 60 and 157 (1.05 vs. 1.06 and 1.05 vs. 1.01 mg/L, respectively; *P* ≥ 0.43). No effects of ZNTRT, ENERGY, or the interaction were observed for liver Mn or Zn (*P* ≥ 0.11). No ZNTRT × ENERGY or ENERGY effects were noted for liver Cu (*P* ≥ 0.20). Liver Cu concentrations tended to be affected by ZNTRT (*P* = 0.07) on d 97 where ZNBLD was lesser than CON (*P* = 0.04) and on d 153 where INZN was lesser (*P* = 0.04) and ZNBLD tended to be lesser (*P* = 0.06) than CON.

**Table 5.** Dietary Zn and energy concentration influence on plasma trace mineral and blood metabolites concentrations analyzed as repeated measures during the finishing period

	CON^*a*^		INZN^*a*^		ZNBLD^*a*^			*P*-value		
Item^*b*^	LE^*c*^	HE^*c*^	LE^*c*^	HE^*c*^	LE^*c*^	HE^*c*^	SEM	ZNTRT	ENERGY	ZNTRT × ENERGY
Steers (*n*)	6	6	6	6	6	5				
Plasma										
Cu^*d*^, mg/L	1.00	1.03	1.01	1.09	0.96	1.00	0.050	0.37	0.22	0.88
Zn^*d*^,	1.21^c^	1.23^c^	1.34^bc^	1.49^a^	1.42^ab^	1.32^bc^	0.047	0.01	0.54	0.04
NEFA^*e*^, mEq/L	193	185	192	178	190	165	13.2	0.67	0.15	0.79
SUN^*f*^, mg/dL	10.1	10.7	10.9	10.9	10.6	10.7	0.70	0.75	0.71	0.89

^*a*^Control (CON) received no supplemental Zn (diets analyzed 33 mg Zn/kg DM); Inorganic Zn (INZN) diet was CON + 120 mg supplemental Zn/kg DM as ZnSO_4_; Zinc blend (ZNBLD) diet was CON + 60 mg supplemental Zn/kg DM as ZnSO_4_ and 60 mg supplemental Zn/kg DM as Zn-amino acid complex (Availa-Zn; Zinpro Corporation, Eden Prairie, MN).

^*b*^Analyzed as repeated measures (d 60, 90, 122, and 157) utilizing the compound symmetry covariate structure to achieve the lowest Akaike information criterion value, values shown are overall repeated measures means.

^*c*^LE: low energy diet formulated to target ~1.6 kg ADG; HE: high energy diet formulated to target ~2.0 kg ADG.

^*d*^There were no ZNTRT × ENERGY × time or ZNTRT × time effects (*P* ≥ 0.19) but an ENERGY × time interaction for plasma Cu (*P* = 0.02). No interactions with time were detected for plasma Zn (*P* ≥ 0.24). Results with differing superscripts are different (a,b; *P* ≤ 0.05).

^*e*^NEFA concentrations differed over time (*P* = 0.0001) and were greater on d 60 than all other time points (*P* ≤ 0.0001; 232 mEg/L), while d 90, 122, and 157 did not differ (*P* ≥ 0.12; 168, 165, and 176 mEg/L, respectively).

^*f*^Serum urea nitrogen concentrations (10.2, 10.9, 10.6, and 10.8 mg/dL for d 60, 90, 122, and 157, respectively) tended to differ over time (*P* = 0.10), where d 60 was lesser than d 90 and 157 but did not differ from d 122 while d 90, 122, and 157 did not differ from each other.

**Table 6.** Dietary Zn and energy concentration influence on liver trace mineral concentrations and liver and muscle relative mRNA expression for MT1A and ZIP14

	CON^*a*^		INZN^*a*^		ZNBLD^*a*^			*P*-value		
Item	LE^*b*^	HE^*b*^	LE^*b*^	HE^*b*^	LE^*b*^	HE^*b*^	SEM	ZNTRT	ENERGY	ZNTRT × ENERGY
Steers (*n*)	6	6	6	6	6	5				
Liver										
Cu, mg/kg DM										
d 97	373	342	270	345	260	292	33.3	0.07	0.37	0.30
d 153	305	277	203	267	214	260	26.9	0.07	0.22	0.20
Mn, mg/kg DM										
d 97	7.8	8.7	7.7	8.5	8.2	8.7	0.56	0.85	0.11	0.94
d 153	9.2	8.6	8.0	8.9	9.4	9.1	0.40	0.14	0.96	0.16
Zn, mg/kg DM										
d 97	104	125	117	125	110	106	7.9	0.25	0.22	0.34
d 153	106	103	113	112	116	108	4.4	0.14	0.27	0.67

^*a*^Control (CON) received no supplemental Zn (diets analyzed 33 mg Zn/kg DM); Inorganic Zn (INZN) diet was CON + 120 mg supplemental Zn/kg DM as ZnSO_4_; Zinc blend (ZNBLD) diet was CON + 60 mg supplemental Zn/kg DM as ZnSO_4_ and 60 mg supplemental Zn/kg DM as Zn-amino acid complex (Availa-Zn; Zinpro Corporation, Eden Prairie, MN).

^*b*^LE: low energy diet formulated to target ~1.6 kg ADG; HE: high energy diet formulated to target ~2.0 kg ADG.

**Figure 1.**
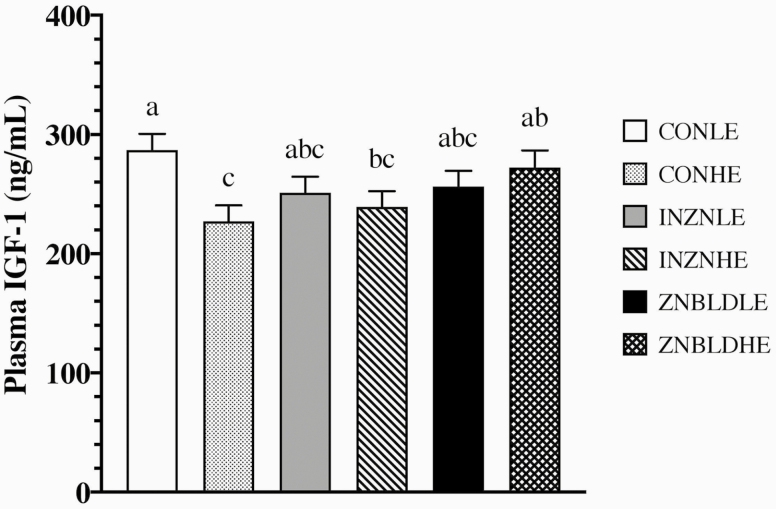
The effect of either no supplemental Zn (33 mg Zn/kg DM; CON) or 150 mg Zn/kg DM (CON + 120 mg Zn/kg DM as ZnSO_4_ [INZN] or CON + 60 mg Zn/kg DM as ZnSO_4_ and 60 mg Zn/kg DM as Availa-Zn [ZNBLD; Zinpro Corporation, Eden Prairie, MN]) in combination with diets formulated to achieve ~1.6 kg ADG (LE) or ~2.0 kg ADG (HE) on plasma insulin-like growth factor 1 (IGF-1) concentrations. Data analyzed as repeated measures from d 90, 122, and 157; ZNTRT × ENERGY; *P* = 0.04. Differing superscripts (a,b,c) indicate means differ (*P* ≤ 0.05).

No ZNTRT, ENERGY, ZNTRT × ENERGY, or interactions with time were noted for SUN or plasma NEFA concentrations (*P* ≥ 0.15; Table 5). Serum urea nitrogen concentrations (10.2, 10.9, 10.6, and 10.8 mg/dL for d 60, 90, 122, and 157, respectively) tended to differ over time (*P* = 0.10), where d 60 was lesser than d 90 and 157 but did not differ from d 122 while d 90, 122, and 157 did not differ from each other. Plasma NEFA concentrations (232, 168, 165, and 176 mEq/L for d 60, 90, 122, and 157, respectively) differed over time (*P* = 0.0001), where d 60 was greater than all other time points. No ZNTRT × ENERGY × time effect was noted for plasma IGF-1 concentrations (*P* = 0.30). A ZNTRT × ENERGY effect was noted for plasma IGF-1 concentrations (*P* = 0.04) where CON LE was greater than CON HE and INZN HE (*P* ≤ 0.02) and ZNBLD HE was greater than CON HE (*P* = 0.04; Figure 1). Additionally, an ENERGY × time effect was noted for plasma IGF-1 concentrations (*P* = 0.0001) where on d 122 LE was greater than HE (*P* = 0.0039) while on d 90 and 157 ENERGY did not differ (*P* ≥ 0.20). A tendency for a positive correlation was noted between plasma IGF-1 concentrations and liver MT1A mRNA expression on d 90 (*r* = 0.33; *P* = 0.06), which can be interpreted as when plasma IGF-1 concentrations increase MT1A expression decreases, but no correlation was detected on d 153 (*r* = −0.12; *P* = 0.50).

### Gene Expression

**Figure 2.**
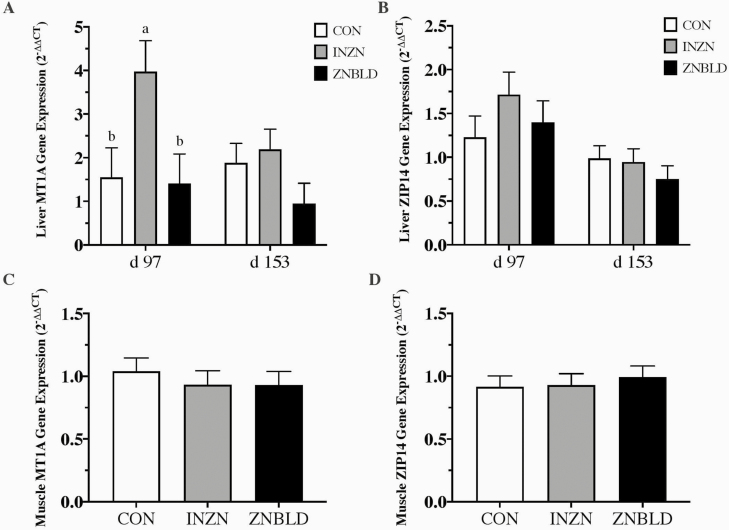
The effect of either no supplemental Zn (33 mg Zn/kg DM; CON) or 150 mg Zn/kg DM (CON + 120 mg Zn/kg DM as ZnSO_4_ [INZN] or CON + 60 mg Zn/kg DM as ZnSO_4_ and 60 mg Zn/kg DM as Availa-Zn [ZNBLD; Zinpro Corporation, Eden Prairie, MN]) on (A) Metallotheionein 1A (MT1A) liver gene expression on d 97 and d 153, (B) Zrt- and Irt-like protein 14 (ZIP14) liver gene expression on d 97 and d 153, (C) Metallotheionein 1A (MT1A) muscle gene expression on d 97, and (D) Zrt- and Irt-like protein 14 (ZIP14) muscle gene expression on d 97. Differing superscripts (a,b) indicate means differ within a day (*P* ≤ 0.05). No ZNTRT × ENERGY or ENERGY effects were detected for any tissue gene expression measures (*P* ≥ 0.32).

No ZNTRT × ENERGY or ENERGY effects were detected for ZIP14 or MT1A in d 97 or 153 liver or d 97 muscle (*P* ≥ 0.32). In d 97 liver, ZNTRT affected MT1A expression (*P* = 0.03) where INZN was greater than ZNBLD or CON (*P* ≤ 0.03) while ZNBLD and CON did not differ (*P* = 0.88; Figure 2A). There were no effects of ZNTRT (*P* ≥ 0.15) on liver MT1A on d 153 (Figure 2A), liver ZIP14 on d 97 and 153 (Figure 2B), or muscle MT1A (Figure 2C) or ZIP14 on d 97 (Figure 2D).

## DISCUSSION

Zinc is an integral component in multiple growth processes including DNA and protein synthesis and the growth hormone and IGF-1 pathway (Clifford and MacDonald, 2000; Maret, 2013). Studies in late-stage finishing steers have noted positive performance outcomes due to Zn supplementation (as Zn-amino acid complex; Zn-AA) above NASEM (2016) recommendations, when fed in tandem with the β-adrenergic agonist ractopamine hydrochloride (Genther-Schroeder et al., 2016a, 2016b). Carmichael et al. (2018) reported Zn and N retention were strongly positively correlated in finishing steers. It is possible that during periods of rapid growth, such as those induced by feeding β-AA or more energy dense diets, cattle require additional Zn to support protein accretion.

During the initial 60 d LE period, all steers exceeded expected performance and supplementation of Zn tended to increase BW during this time when compared with un-supplemented steers. Using similar supplemental Zn concentrations the growth response of steers in the 42–86 d prior to β-AA supplementation has been mixed, with no effect on BW (Genther-Schroeder et al., 2016a) or slight increases in BW (Genther-Schroeder et al., 2016b). Growing beef heifers fed a corn-silage based diet supplemented with 25 mg Zn/kg DM as either Zn oxide or Zn methionine had increased gain and feed efficiency during the initial 56 d on trial compared to non-supplemented heifers (Spears, 1989). It was suggested the growth response due to supplemental Zn was because non-supplemented diets were marginally Zn deficient (23 mg Zn/kg DM; Spears, 1989). The CON diet in the present study analyzed 33 mg Zn/kg DM, close to the NASEM (2016) requirement of 30 mg Zn/kg DM, which may have been inadequate to support rates of growth achieved early in the trial. Additionally, the source of Zn did not differentially affect steer performance during the initial growing period of the present study.

The HE diet utilized in the present study was designed to elicit ADG improvements over LE-fed steers that were similar or in excess of the ADG response typically observed during ractopamine hydrochloride supplementation (Lean et al., 2014). The introduction of higher concentrate diets during transition creates many challenges for the ruminant gastrointestinal tract (Dixon and Stockdale, 1999), but improvements in ADG and G:F during the first few weeks following transition suggests steers receiving ZNBLD were better able to adapt to these changes. Zinc is involved in the intestinal mucosal function and epithelial integrity (Alam et al., 1994; Rodriguez et al., 1996; Sturniolo et al., 2001). Highly fermentable diets increase the concentration of short-chain fatty acids in the rumen, effectively decreasing rumen pH and challenging metabolism and intracellular homeostasis of the rumen epithelium (Penner et al., 2011). The effect of Zn supplementation on ruminant gut barrier function does not appear to have been well studied to date, but in swine supplementing 200 mg Zn/kg DM as Zn-amino acid complex improved gut integrity during a 7 d heat stress event (Sanz Fernandez et al., 2014). Further examination is needed to determine the role, if any, ZNBLD may play in protection against gastrointestinal stress in cattle. It is interesting to note that the improvement in overall ADG in HE vs. LE steers was only 2% within unsupplemented CON, while INZN and ZNBLD increased ADG by 5% and 13%, respectively, as energy density of the diet increased. The results of the current study suggest there is a relationship between supplemental Zn and dietary energy, however further research is needed to fully elucidate the nature of this relationship.

The Zn importer ZIP14 is one mechanism allowing cellular Zn influx, and ZIP14 knock out mice exhibit stunted growth and impaired gluconeogenesis, potentially due to the role of Zn in modulation of G-coupled protein receptor signaling pathways critical to growth (Hojyo et al., 2011). However, increasing dietary energy did not affect ZIP14 expression in the present study, and the lack of change in ZIP14 expression due to supplemental Zn is similar to previous reports (Lichten et al., 2009; Aydemir and Cousins, 2018).

Free Zn (not bound to a protein; Krężel and Maret, 2016) induces MT mRNA expression, but the MT protein has a greater binding affinity for Cu, resulting in Cu bound to MT in the enterocyte being lost in feces as enterocytes are sloughed off (Cousins, 1985). Though intestinal MT was not assessed, liver Cu concentrations in the present study decreased due to supplemental Zn but remained adequate (Kincaid, 2000) regardless of ZNTRT. The status of some trace minerals (e.g., Cu and Se) in cattle can be assessed according to liver concentrations; however, supplementing Zn at five times current NASEM (2016) recommendations had no effect on liver Zn concentrations, supporting the assertation that liver Zn is insufficiently sensitive to distinguish among cattle with adequate or greater Zn status. Therefore, MT1A was examined as a potentially more sensitive measure for Zn status and availability in ruminants. Interestingly, liver MT1A mRNA expression on d 97 was greater for INZN compared with ZNBLD and CON, and previous studies have shown an increase in hepatic MT due to increased supplemental Zn in chicks (as Zn acetate, Zn methionine, and Zn proteinate; Cao et al., 2002), Holstein calves (Zn proteinate and ZnSO_4_; Wright and Spears, 2010) and sheep (Zn lysine; Rojas et al., 1996). However, this difference was null by d 153, suggesting possible changes in absorption and excretion after long periods of greater Zn supplementation, such as that in rats where excretion and absorption shift to maintain homeostatic Zn accretion above requirements (Weigand and Kirchgessner, 1978). Metallothionein induction by Zn is accomplished through metal transcription factor-1 (MTF-1) and is only induced by free Zn which indicates accumulation of Zn in the cytosol (Fukada and Kambe, 2014; Kimura and Kambe, 2016). Previous research results indicate some Zn-amino acid complexes may be absorbed through amino acid transporters (Glover and Hogstrand, 2002; Sauer et al., 2017) and since liver Zn concentrations did not differ due to ZNTRT, the Zn-AA source of Zn may be bound to an amino acid in the liver unavailable for recognition by MTF-1, while ZnSO_4_ may exist in the liver as free Zn. Further work should be conducted to better understand differences between Zn sources in post-absorptive metabolism as well as source effects on putative Zn biomarkers in ruminants.

Plasma Zn was adequate in all treatments (Kincaid, 2000), and only INZN-HE and ZNBLD-LE had greater plasma Zn concentrations than unsupplemented CON steers. Zinc source solubility and absorption and physiological utilization can be impacted by the diet (Spears, 1989; Garg et al., 2008; Faulkner et al., 2017; VanValin et al., 2018), and may have influenced the differences in plasma Zn concentrations observed herein. Carmichael et al. (2019) found that Zn retention was greater in steers receiving a low energy, high fiber diet vs. those receiving a high concentrate diet regardless of Zn supplementation and that supplementing the same blend of Zn sources as those in the current study (60 mg Zn/kg DM as ZnSO_4_ and 60 mg Zn/kg DM as Zn-AA) also increased Zn retention. Overall, if animals are not Zn deficient plasma Zn has limited use as a Zn biomarker as it does not appear to be related to whole animal Zn retention.

Steers receiving supplemental Zn exhibited limited advantages in a final performance in the present study. This is in accordance with previous studies where supplementing similar concentrations of Zn as Zn oxide to finishing heifers did not improve final BW or ADG (Van Bibber-Krueger et al., 2017, 2019). Van Bibber-Krueger et al. (2017) similarly saw no differences in plasma urea N when 100 mg Zn/kg DM as Zn oxide was supplemented to finishing heifers. Zinc has been shown to increase N retention in finishing steers (Carmichael et al., 2018) and is integral for N utilization in other mammals (Oberleas and Prasad, 1969; Greeley et al., 1980). Furthermore, N retention has been shown to improve with β-AA supplementation and use of anabolic implants in Holstein steers, but no differences in plasma urea N were detected due to either treatment (Walker et al., 2007). Neither dietary energy nor supplemental Zn affected SUN in the present study and collectively SUN do not appear to be reflective of increased N retention (protein accretion) in feedlot steers.

It is well recognized that increasing dietary energy increases cattle growth rate (Geay, 1984; Owens et al., 1993, 1995). In the present study, HE increased G:F, HCW, MS, BF, and YG regardless of ZNTRT when compared with LE. The anabolic hormone IGF-1 is heavily involved in growth and maintenance of muscle (Estívariz and Ziegler, 1997; Maggio et al., 2013) and in the current study LE generally had greater plasma IGF-1 with no ZNTRT effects noted. However, within HE ZNBLD had greater IGF-1 than CON with the inorganic treatment being intermediate. Previous research has shown that IGF-1 inversely regulates MT expression, and since downregulation of MT would increase the amount of free Zn available for protein incorporation, it was suggested that Zn may be involved in balancing remodeling skeletal muscle (Latres et al., 2005). Zinc plays an integral role in IGF-1 activity, inhibiting tyrosine phosphatase activity, therefore increasing activity of the p70 S6 kinase, MAPK, and EGF pathways (Haase and Maret, 2003) and when induced by IGF-1 result in hypertrophy of skeletal muscle. A tendency for a positive correlation was detected between plasma IGF-1 concentration on d 97 and MT1A ΔCT values on d 90 of the current study, which is interpreted to mean that when MT1A concentrations decreased IGF-1 expression increased, but this correlation was not observed on d 153. Further work should be conducted to determine the relationship between circulating IGF-1 and MT1A gene expression in ruminants.

Previous research in ruminants and humans has shown that IGF-1 concentrations will change depending on nutritional status (Estívariz and Ziegler, 1997; Maggio et al., 2013). Following 32 d after an anabolic implant circulating concentrations of IGF-1 in the current study were increased in LE compared with HE on d 122. Studying the effects of an estrogenic implant or a low-energy diet in swine, Lee et al. (2005) found that implant increased circulating IGF-1 concentrations but was unaffected by the low energy diet. It is well established that following administration of an anabolic implant circulating IGF-1 concentrations will increase in cattle (Johnson et al., 1996, 1998; Walker et al., 2007). Additionally, Hutcheson et al. (1997) noted that steers receiving estrogenic or combination implants decrease NEg requirements ~19% and increase daily protein accretion over non-implanted steers. According to the current study, it appears steers receiving LE were more receptive to the action of implants ability to increase circulating IGF-1 concentrations, and while LE did not surpass growth induced by HE following implant on d 90, there was no advantage of HE over LE for ADG during d 122–158. Further research should be conducted to determine the impacts of combination anabolic implants on circulating IGF-1 concentrations in feedlot cattle receiving diets differing in energy content.

Increasing dietary energy improved performance measures in finishing cattle regardless of Zn supplementation strategy; however, targeted improvements in growth rate of HE over LE were not achieved. Collectively, increasing supplemental Zn during the growing period improved performance, but limited final performance advantages were noted due to increased Zn supplementation concentrations. Differences in liver gene expression of the Zn storage protein metallothionein suggest post-absorption metabolism may differ between ZnSO_4_ and Zn-amino acid complex and future work is needed to better understand this discrepancy between sources.
